# Membrane-based continuous fermentation with cell recycling for propionic acid production from glycerol by *Acidipropionibacterium acidipropionici*

**DOI:** 10.1186/s12934-023-02049-7

**Published:** 2023-03-04

**Authors:** Victor Hugo Cavero-Olguin, Tarek Dishisha, Rajni Hatti-Kaul

**Affiliations:** 1 Division of Biotechnology, Department of Chemistry, Center for Chemistry & Chemical Engineering, Lund University, 124, 221 00 Lund, Sweden; 2 Área de Biotecnología, Instituto de Investigaciones Fármaco Bioquímicas, Facultad de Ciencias Farmacéuticas y Bioquímicas, Universidad Mayor de San Andrés, 3239, La Paz, Bolivia; 3 Department of Pharmaceutical Microbiology and Immunology, Faculty of Pharmacy, Beni-Suef University, Beni-Suef, 62511 Egypt

**Keywords:** Propionic acid, Glycerol, Propionibacteria, Fermentation, Biorefinery

## Abstract

**Background:**

Microbial production of propionic acid (PA) from renewable resources is limited by the slow growth of the producer bacteria and product-mediated inhibition. The present study evaluates high cell density continuous PA fermentation from glycerol (Gly) using *Acidipropionibacterium acidipropionici* DSM 4900 in a membrane-based cell recycling system. A ceramic tubular membrane filter of 0.22 μm pore size was used as the filtering device for cell recycling. The continuous fermentations were run sequentially at dilution rates of 0.05 and 0.025 1/h using varying glycerol concentrations and two different yeast extract concentrations.

**Results:**

PA volumetric productivity of 0.98 g/L.h with a product yield of 0.38 g_PA_/g_Gly_ was obtained with 51.40 g/L glycerol at a yeast extract concentration of 10 g/L. Increasing the glycerol and yeast extract concentrations to 64.50 g/L and 20 g/L, respectively, increased in PA productivity, product yield, and concentration to 1.82 g/L.h, 0.79 g_PA_/g_Gly_, and 38.37 g/L, respectively. However, lowering the dilution rate to 0.025 1/h reduced the production efficiency. The cell density increased from 5.80 to 91.83 g_CDW_/L throughout the operation, which lasted for a period of 5 months. A tolerant variant of *A. acidipropoinici* exhibiting growth at a PA concentration of 20 g/L was isolated at the end of the experiment.

**Conclusions:**

Applying the current approach for PA fermentation can overcome several limitations for process industrialization.

## Background

Propionibacteria are anaerobic, Gram-positive bacteria that besides being used for the production of traditional Swiss-type cheeses and as a probiotic for humans and animals [1, 2], are also an important source of valuable products like vitamin B12, trehalose, flavors, antimicrobial compounds including diketopiperazines, linear- and cyclic peptides, 3-phenyllactic acid and propionic acid [3, 4]. Propionic acid (PA), a C-3 organic acid, is a product of great interest with uses ranging from the food and feed industry, pharmaceuticals, cosmetics, and plastics [5, 6]. Its main uses are as preservative; in the form of calcium, sodium and ammonium salts, for animal feed, grains, and foods; around half of global production of PA goes for cattle feed application [7, 8], while the preservation of baked foods is another major application. PA-based derivatives like 2-aryl propionic acid are frequently prescribed as anti-inflammatory agents [9]. Some of the other value-added uses of PA include its use as an intermediate in the synthesis of ester solvents, flavors and fragrances (like ethyl- and benzyl propionate), thermoplastic polymers (e.g. cellulose propionate), and as a precursor for pesticides and pharmaceuticals (e.g. vinyl propionate) [10–12].

The commercial production of PA still depends on petrochemistry, although there have been ongoing research efforts to establish a competitive bio-based route of production. The fossil-based PA costs around 1 US dollar/kg, while that obtained via microbial fermentation ranges between 1.5–2 USD/kg [5, 8, 13, 14]. The higher cost of the fermentation route is attributed to the feedstock, low process efficiency, and energy-consuming downstream processing [15]. According to economic assessments, it is estimated that PA volumetric productivity in the range of 2 g/L/h, yield of 0.6 g/g (PA/sugar), and titer of 100 g/L are needed for the bioproduction to be economically viable [16–18].

Propionibacteria are the main producers of PA from different carbon sources like glucose, lactose, lactate, xylose, and glycerol (Gly) using a pathway called the Wood-Werkman cycle involving succinate decarboxylation [14, 16, 19–29]. The higher degree of reduction of glycerol (κ = 4.667) compared to glucose, lactose, xylose and lactate (κ = 4), thermodynamically favors the production of the more reduced PA (κ = 4.67) than acetic acid (AA) (κ = 4) as a by-product [12, 20, 22, 30]. In this case, the conversion of glycerol to pyruvate generates more reducing equivalents, driving the metabolic flux towards PA to achieve the co-factor balance. Glucose, on the other hand, generates a lower amount of reducing equivalents than glycerol, and hence acetate production is preferred to generate more ATP for cell growth. Consequently, the PA/AA molar ratio is greatly affected by the carbon source and could vary between 2:1 when lactate is utilized to 30:1 with glycerol [12, 14, 20, 22, 25, 26]. Glycerol is also a major co-product of biodiesel production [31], finds several applications directly after refining, and also serves as an attractive feedstock for other chemical building blocks [32].

The propionic acid fermentation process is slow and limited by product inhibition, resulting in low productivity and yield. Hence, the use of high cell density reactors has been proposed to overcome the limitation. Several studies on enhancing propionic acid production using immobilized cell bioreactors have been reported [23, 25, 29, 33–38], the majority of which utilize fibrous bed reactors. We have also reported sequential batch propionic acid fermentation with cell recycling using free cells of *Acidipropionibacterium acidipropionici*, which were collected by centrifugation after each batch and then used to start a new batch of fermentation [14]. Using a culture medium with 50 g/L glycerol, the cell biomass concentration increased 215 times during 9 batches that reached 21.5 g_CDW_/L, while the productivity increased sixfold to 1.35 g/L.h. By further optimizing the concentrations of carbon and nitrogen sources, the productivity reached 1.63 g/L.h with a final propionic acid concentration of 60 g/L and biomass of 31.28 g/L [26].

Membrane-based cell retention, applied primarily for cell separation during product recovery in biotechnology processes [39, 40], offers a useful alternative for high cell density fermentation without any cell leakage [41]. The latter technique has been used on a lab scale for the production of several metabolites like ethanol, acetone-butanol, citric acid, lactic acid, and mannitol [41–45]. A number of studies have reported PA production from sugars, whey, and lactic acid using a continuous mode of operation with cell recycling [19, 46–49], while only one study employed glycerol as a carbon source achieving productivity of 1 g/L.h with a PA concentration of 10 g/L [11].

The choice of a suitable membrane is important for the success of the application, the important criteria for selection being identified as mechanical strength, resistance to cleaning agents, pore size, and surface charge [50]. A major limitation of the membrane processes is the decrease in permeate flux due to membrane fouling, the extent of which depends on the process operating parameters and the interaction of the membrane with feed components. During the past two decades, there has been increasing interest in ceramic membranes that are based on alumina (Al_2_O_3_), zirconia (ZrO_2_), titania (TiO_2_), or a combination of these materials [29]. The ceramic membranes have several advantages over polymeric membranes including high resistance to aggressive physical and chemical cleaning and corrosion, and inertness to biological components, allowing them to possess high flow capacity, high separation efficiency, and long shelf lives [40, 51, 52].

This report presents a study on continuous PA fermentation involving cell recycling using a ceramic (TiO_2_) membrane filter at varying glycerol and yeast extract (YE) concentrations as carbon and nitrogen sources, respectively.

## Results and discussion

### Batch cultivation

**Fig. 1:**
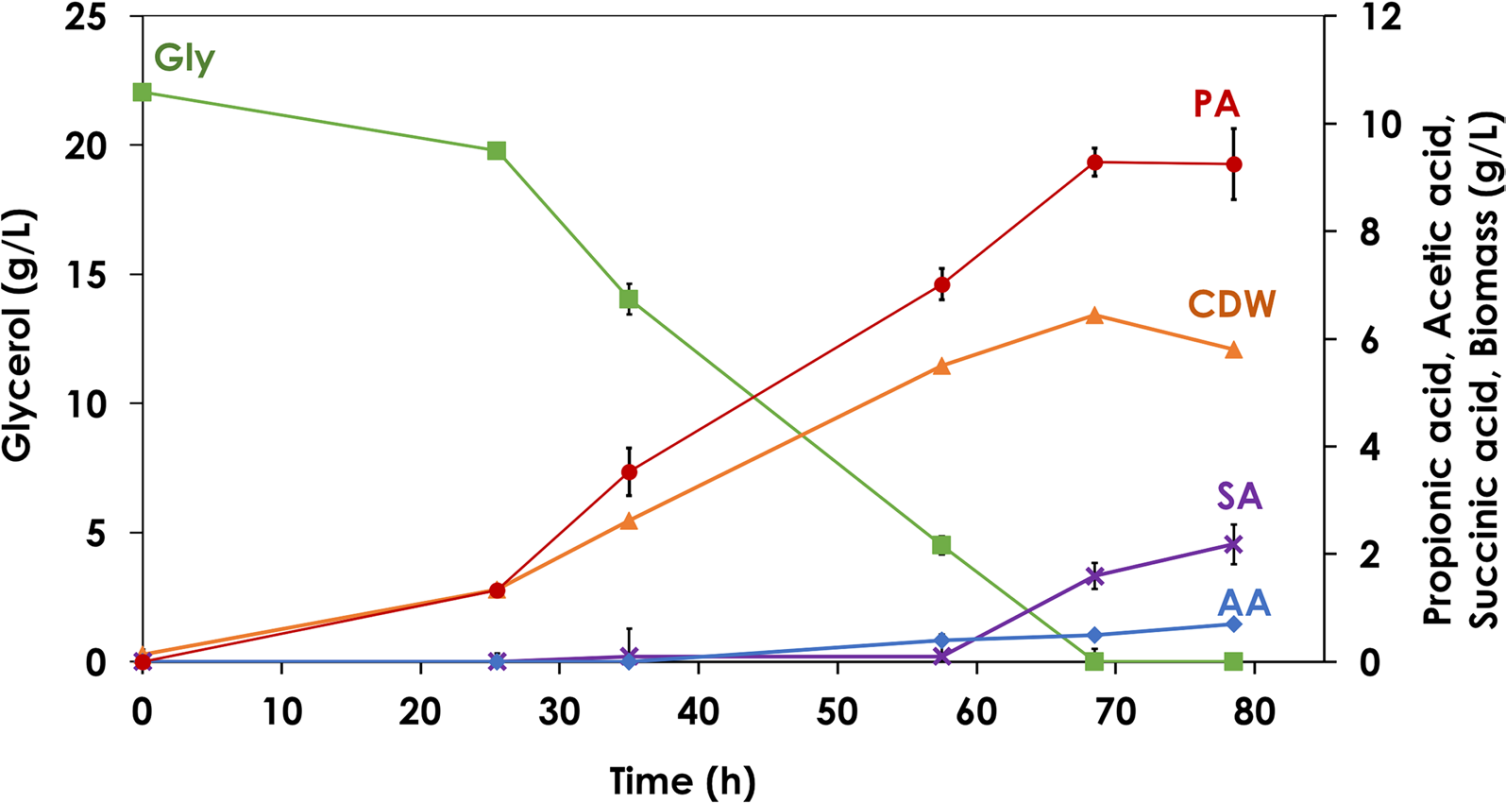
Propionic acid production from glycerol under batch mode of operation. Batch cultivation of *Acidipropionibacterium acidipropionici* with glycerol:yeast extract ratio of 20:10 g/L. The different symbols represent biomass (▲), glycerol (■), propionic acid (●), succinic acid (✕) and acetic acid (♦) concentrations

PA fermentation was started in a batch mode in the bioreactor, for which actively growing cells of *A. acidipropionici* were used to inoculate the basal medium containing 22.04 g/L glycerol and 10 g/L yeast extract. As the growth of propionic acid bacteria obtained from the stock culture at − 20 °C is characterized by a long lag phase (3–4 days in the preculture prepared with ~ 1% inoculum), a 7-day old preculture (with an OD_620nm_ of ~ 7) with cells in the exponential phase, was used as inoculum. Glycerol was depleted in less than 70 h in the batch fermentation, resulting in PA volumetric productivity, yield and titer of 0.14 g/L.h, 0.42 g_PA_/g_Gly,_ and 9.29 g/L, respectively. The cell density increased from 0.13 g_CDW_/L (OD_620nm_ of 0.36) at 0 h to 5.80 g_CDW_/L (OD_620nm_ of 15.85) at the time of glycerol depletion with biomass productivity of 0.09 g_CDW_/L.h (Fig. 1).

### Continuous chemostat with cell retention for propionic acid production

Directly after the batch fermentation, the continuous mode of operation of the bioreactor coupled to the membrane unit was started with the feed set at a constant flow rate of 18 mL/h (D = 0.05 1/h) during 40 days followed by a feed rate of 9 mL/h (D = 0.025 1/h) for 18 days. With the D = 0.05 1/h, the glycerol concentration was increased stepwise from 22.60 g/L to 64.5 g/L, while the effect of doubling the yeast extract concentration (from 10 to 20 g/L) when reaching 64.50 g/L glycerol, was also studied. The latter was based on our earlier studies showing the positive effect of increasing the concentration of nitrogen source on the fermentation kinetics [26].

**Fig. 2:**
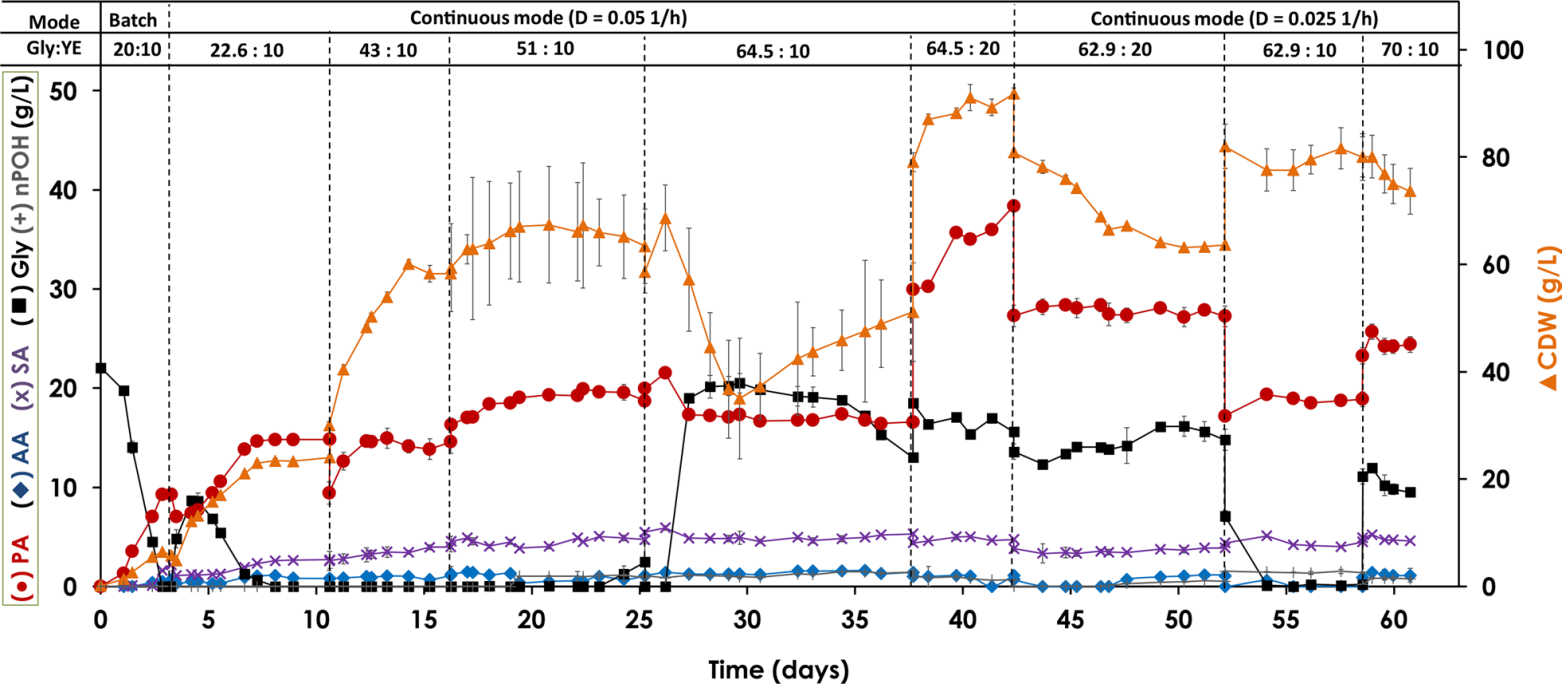
Propionic acid production from glycerol under continuous mode of operation with cell recycling. Continuous propionic acid production from glycerol with cell recycling via a tangential flow filtration module with a ceramic membrane filter. The concentrations of biomass (▲), propionic acid (●), glycerol (◼), acetic acid (♦), succinic acid (✕), and n-propanol ( +) are presented during two different dilution rates. The first five stages correspond to D = 0.05 and the last three stages correspond to D = 0.025, with different medium composition (Gly:YE ratio of 22.60:10, 43.27:10, 51.40:10, 64.50:10, 64.50:20, 62.90:20, 62.90:10, 70:10 g/L, respectively)

As seen in Table 1, the first condition used, i.e. with 22.60 g/L glycerol and 10 g/L yeast extract resulted in over a fivefold increase in productivity (0.74 g/L.h) and about 1.6 fold higher PA concentration (14.80 g/L) as compared to the batch fermentation (Fig. 1), as a direct effect of the retention of the cells by the microfiltration membrane. Raising the substrate concentration from 22.60 g/L to 51.40 g/L led to further enhancement in productivity (0.98 g/L.h) and product concentration (19.56 g/L) with complete consumption of glycerol. With a further increase in glycerol concentration to 64.50 g/L, only 79.83% was consumed and there was a drop in both productivity (0.85 g/L.h) and product concentration (16.57 g/L) (Fig. 2). The product yield increased from 0.42 g_PA_/g_Gly_ to 0.66 g_PA_/g_Gly_ when going from batch to continuous mode of operation with 22.60 g/L glycerol and was maintained in the range of 0.33–0.38 g/L with increasing glycerol concentration to 64.50 g/L (Table 1).

**Table 1:** Propionic acid production under continuous operation with cell recycling performed at different dilution rates (D), C- (glycerol) and N-source (yeast extract) concentrations

Dilution rate (D)^a^	0.05 1/h					0.025 1/h		
Glycerol (g/L) : YE (g/L)^b^	**22.60:10**	**43.27:10**	**51.40:10**	**64.50:10**	**64.50:20**	**62.90:20**	**62.90:10**	**70.00:10**
*P* (g/L)	14.78 ± 0.08	14.45 ± 0.40	19.35 ± 0.40	16.94 ± 0.34	36.25 ± 1.47	27.82 ± 0.48	18.77 ± 0.20	24.27 ± 0.12
*X* (g_CDW_/L)	23.44 ± 0.43	58.90 ± 0.84	61.57 ± 8.97	49.12 ± 7.99	90.09 ± 1.44	63.56 ± 0.35	77.49 ± 3.79	74.81 ± 3.84
*Q*_*P*_ (g/L/h)	0.73 ± 0.00	0.72 ± 0.02	0.98 ± 0.01	0.85 ± 0.02	1.82 ± 0.09	0.69 ± 0.01	0.47 ± 0.01	0.62 ± 0.02
*Y*_*P/S*_ (g_P_/g_S_)	0.65 ± 0.02	0.33 ± 0.01	0.38 ± 0.01	0.37 ± 0.02	0.75 ± 0.06	0.57 ± 0.01	0.30 ± 0.01	0.41 ± 0.02

Production of propoinic acid from glycerol under continuous mode of operation with cell recycling using tangential flow ceramic membrane. ^a^Different dilution rates were tested with ^b^different glycerol and yeast extract (YE) concentrations in the feeding solution. Biomass (*X*), propionic acid (*P*) concentrations, volumetric productivity (*Q*_*p*_) and product yield (*Y*_*P/S*_) are presented

The relatively low product yield was most likely due to the carbon source being used more for biomass formation as seen in a substantial increase in the cell mass concentration, especially up to 43.27 g/L glycerol feed (Table 1). This was also reflected in the increase in volumetric biomass productivity from 0.15 g_CDW_/L.h at 22.60 g/L glycerol to 0.27 g_CDW_/L.h at 43.27 g/L and then dropping to 0.09 and 0.08 g_CDW_/L.h at 51.40 and 64.50 g/L glycerol, respectively. These observations were attributed to nutrient limitation at higher substrate concentrations; raising the yeast extract concentration to 20 g/L with glycerol being maintained around 64.50 g/L, led to enhanced fermentation kinetics, especially PA productivity which reached 1.82 g/L.h and a final PA concentration of 38.37 g/L. This was concomitant with a drastic increase in cell concentration up to 90.09 g_CDW_/L with volumetric biomass productivity of 0.11 g_CDW_/L.h (Table 1 and Fig. 2). In spite of this, only 75.80% of the initial glycerol amount was consumed. The high biomass concentration resulted in membrane clogging, and on the 42nd day, the feed was temporarily stopped to enable bleeding of the culture. Increasing the yeast extract concentration to 30 g/L was reported earlier to further enhance the fermentation rate [26], however, this was not considered in the present experiment to avoid further increase in the biomass concentration and also to reduce the fluctuations in the product levels and productivity in the system. Instead, it is more important to maintain a high density of metabolically active cells, which is achieved by introducing cell bleeding at definite time intervals.

Continuing fermentation with glycerol and yeast extract concentrations of 62.90 and 20 g/L, respectively, at a lower feed rate of 9 mL/h (equivalent to a dilution rate of 0.025 1/h) did not have a favorable effect on the volumetric productivity and PA titer, especially when the yeast extract concentration was lowered to 10 g/L (Table 1 and Fig. 2). The cell biomass concentration was decreased due to bleeding but then stabilized even when decreasing the nitrogen source. PA production parameters were then partly regained by increasing the glycerol concentration to 70.00 g/L (Table 1). Glycerol consumption at 62.90:20 and 70:10 g/L Gly:YE concentrations was 76.47% and 86.42%, respectively. The reactor was then kept working for an additional three-month period in the same manner with glycerol and yeast extract concentrations ranging between 20:10 and 70:10 g/L at dilution rates of 0.05 and 0.025 1/h (data not shown). This was done primarily to evaluate the stability of the system.

Summarizing the observations from Table 1, it is evident that PA production was more favorable at a higher dilution rate (0.05 1/h), a decrease in cell growth rate led to an increased carbon flux towards the product, and an increase in nitrogen concentration had a dramatic effect on improving the productivity and product yield. Lowering the dilution rate lowered the PA production levels and kinetics but did not shift the carbon flux to biomass formation.

AA and succinic acid (SA) were formed as main byproducts with rather stable levels during the entire period, which were in the range of 0.30–1.61 g/L and 1.10–5.30 g/L, respectively, and reaching maximum AA:PA and SA:PA ratios of 0.10 and 0.31, respectively (Fig. 2). Formation of these by-products, even though not significantly high, impacts the PA yield and also adds to downstream processing costs. Low levels of AA formed are characteristic of PA production from glycerol, as also reported earlier, e.g. for *P. thoenii* (AA:PA ratio of 0.01) [11] and *P. freudenreichii* subsp. *shermanii* (AA:PA ratio of 0.03) [53﻿]. In contrast, batch cultivations with *A. acidipropionici* using glucose–xylose mixture at a ratio of 3:1 resulted in AA:PA ratio of 0.28 [19]. Also *P. freudenreichii* subsp. *shermanii* grown on glucose gave AA:PA and SA:PA ratios of 0.39 and 0.21, respectively, while with the use of glycerol the ratios were 0.03 and 0.1, respectively [53]. *A. acidipropionici* grown on sugar-cane molasses, a low-cost industrial waste (40 g/L) and lactate (30 g/L) gave AA:PA of 0.37 and 0.27, respectively [20], while no AA was detected with glycerol used at 20 g/L [20].

The slightly higher concentrations of AA and SA in the membrane bioreactor may indicate some inhibition of the metabolic pathway under the conditions in the reactor [54]. In an earlier report, the use of another polyol, sorbitol, in sequential batches with *A. acidipropionici* provoked a decrease in AA and PA levels from 3.3 to 2.0 g/L and 39.5 to 34.4 g/L, respectively, while SA accumulation was increased from 6.1 to 14.8 g/L [55].

In principle, there are no limits to increasing the dilution rates with cell-retention systems, since cell washout is prevented by the filtering device. The accumulated cells could stand high loads of substrate while possible inhibitory products are constantly removed [56]. In the present work, the production of PA was enhanced by adjusting the concentrations of carbon and nitrogen sources and not by the retention time. Our observations seem to be in agreement with an earlier report showing no significant differences in the production of biomass, mannitol, lactic, and acetic acid with *Leuconostoc citreum* in a ceramic membrane filter fermentation system at different dilution rates [43]. During continuous fermentation for lactic acid production using *Bacillus coagulans* with a ceramic filter device, an increase in glucose concentration in the feed from 50 to 70 g/L at 0.20 1/h resulted in the same concentration of lactic acid in the product stream along with 20 g/L of residual sugar [44].

The production parameters obtained in this work were higher than many of the reported data using immobilized cells or other forms of high-cell density systems for PA production [14, 25, 26] (Table 2). With respect to cell retention using a filtration system for PA production, the first report made use of a spin filter in a continuous mode of operation for fermentation of lactose by *A. acidipropionici* at a dilution rate of 0.05 1/h in a 7 L bioreactor, where 50% of the cells were retained obtaining almost four-fold enhancement of the productivity reaching 0.90 g/L.h compared to that in conventional batch fermentation [46], very similar to the fivefold increase in productivity at the same dilution rate obtained in the first continuous stage in the present work. Co-production of PA and succinic acid by *A. acidipropionici* in a semi-continuous fermentation with a 0.20 μm membrane module for separation in a recycling loop coupled with a chromatography device, gave a titer of 62.22 g/L and productivity of 0.43 g/L.h, an increase by 65% [57]. No contamination as well as no leakage of cells into the permeate was observed during the entire process.

**Table 2:** Comparison of propionic acid production parameters obtained in high-cell density systems of *A. acidipropionici* established by different means

Means for high-cell density	Mode of operation	Carbon source, g/L	Q_p_, g/L.h	Y, g_P_/g_S_	Titer, g/L	References
Immobilization on polyethyleneimine coated Poraver beads	Batch	Glycerol, ~ 85 g/L	0.35	0.47	35.23	[25]
Immobilization on polyethyleneimine coated Poraver beads	Continuous	Glycerol, 30 g/L	1.44	0.69	14.38	[25]
Cell recycling	Sequential batch	Glycerol, ~ 50 g/L	1.42	0.63	27.3	[14]
Cell recycling	Sequential batch	Glycerol, 90 g/L	1.63	0.59	ca. 45	[26]
Recycling 10% of the culture	Cyclic batch	Glycerol, 90 g/L	0.53	0.75	42.5	[26]
Spin filter-assisted cell retention	Continuous	Lactose, 50 g/L	0.9	0.40	18.5	[46]
Spin filter-assisted cell retention	Continuous	Whey, 30–35 g/L lactose concentration	0.72	0.54	14.5	[60]
Spiral-membrane module	Semi-continuous	Glucose and glycerol, 100 g/L in total	0.43	-	62.22	[57]
Ultrafiltration module	Continuous	Whey, 25 g/L lactose concentration	2.14	0.43	7	[49]
Ultrafiltration module	Continuous	Glucose/xylose, 50 g/L in total	2.2	0.42	16.2	[19]
Membrane-assisted cell recycling	Continuous	Glycerol, 64.50 g/L	1.82	0.75	36.25	This work

While the accumulated cell concentration reached ~ 6 g_CDW_/L during the batch fermentation, the cell density during the continuous fermentations with the membrane filter device ranged from 23.44 to 90.09 g_CDW_/L (Table 1 and Fig. 2). According to Chang et al. [58], a tenfold density of a batch culture is accepted as high-cell density [58], which implies that the high-cell density state with ~ 59 g_CDW_/L was reached already at Gly:YE concentrations of 40:10 g/L. Nonetheless, uneven rates between the inflow and the outflow occurred during the entire fermentation.

The system was highly affected by the retained cells on the membrane filter, possible cake formation, and increased viscosity of the fermentation broth, leading to a decrease in the filtration rate as also reported earlier for lactic acid fermentation using *B. coagulans* and *Lactococcus lactis* ssp. *lactis*, respectively [44, 59]. Cake formation was found to be prevented when the recycling pump was operated at a lower velocity than that needed for the critical flux through the membrane when lactic acid fermentation lasted 3–5 days and cell concentration was 1.10–3.10 g/L [59], far lower values than the respective ones in the present work. Membrane fouling and the need for high pressures for the operation could be the main bottlenecks when using membrane filters with high cell densities and even with high molecular weight products. For example, the performance of the system involving the cultivation of *Saccharomyces cerevisiae* with an integrated tangential filtration device was affected by the accumulation of polymeric substances onto the surface of the filter [55]. Other studies showed that materials like polyvinylidene difluoride had antifouling properties when used for pyruvic acid production by *Torulopsis glabrata* [42].

The membrane filter retained both metabolically active and inactive cells, the proportions of which would determine how efficiently the substrate is metabolized. Hence understanding the physiological and metabolic state of the cell populations during long-term operation of the reactor could provide clues for the optimal functioning of PA fermentation, and prevent accumulation of unproductive cells and possible inhibitory substances.

### Isolation of acid-tolerant strain

Continuous fermentation over a long period in the membrane-integrated bioreactor exerts extensive stress on the cells which are subjected to mechanical stress due to the constant shearing through pumping and high pressures. The cell suspension, withdrawn from the bioreactor after 5 months of operation, showed growth in media with initial pH adjusted to 7.0, 6.0 and 5.0, respectively, in serum bottles, and reached a final cell density of OD_620_ > 7.0 after three weeks. Cells obtained from the pH-5.0 serum bottle were transferred to media with initial PA concentrations of 10–50 g/L at pH 7. Cell growth with OD_620_ > 7.0 was obtained only in media with 10 and 20 g/L of PA, in contrast to the wild type *A. acidipropionici* DSM 4900 that showed no visible growth at an initial PA concentration of 10 g/L [28]. The adapted cells were then transferred to agar plates with 20 g/L PA at pH 5 to obtain colonies. A colony was isolated and its growth was confirmed in the medium with 20 g/L glycerol and further supplemented with 20 g/L PA at pH 5. The culture was stored in a 50% glycerol medium at − 20 °C for further use.

## Conclusions

The efficient performance of the ceramic membrane filter was demonstrated during a long fermentation period. Significantly higher production efficiency was obtained in comparison with previous reports. Nonetheless, such a system would greatly benefit from the use of a sensor for automated control of fouling on the filter surface and the volume in the reactor vessel to enhance the stability and performance for long-term fermentations and avoid clogging of membranes that leads to perturbation of the fermentation process. With a more stable system, the possibility of using crude glycerin from the biodiesel industry will result in better management of agro-industrial residues. The concomitant adaptation of the strain exposed to mechanical stress and also exposure to high concentrations of glycerol and PA made possible easy isolation of a tolerant variant for further investigation.

## Methods

### Chemicals

Glycerol (99%), ammonium hydroxide solution (28%), and L-cysteine HCl anhydrous (98%) were products of Sigma-Aldrich (St Louis, MO, USA). Bacto yeast extract (YE) was procured from Difco (BD laboratories, Detroit, MI, USA) and phosphate buffer salts from Merck (NJ, USA).

### Microorganism and culture medium

*A. acidipropionici* DSM 4900 was used in the present study. For preculture preparation, the basal culture medium consisted of 10 g/L yeast extract, 20 g/L glycerol, 0.25 g/L cysteine HCl, 2.5 g/L K_2_HPO_4_ and 1.5 g/L KH_2_PO_4_, pH 7.0 adjusted using ammonium hydroxide [14]. The medium (90 mL) was prepared in 100 mL serum bottles, boiled, flushed with oxygen-free nitrogen, sealed, and then autoclaved at 121 °C for 20 min. The bottles were then allowed to cool and used for pre-culture preparation. For bioreactor experiments, the same culture medium was used with varying concentrations of carbon- and nitrogen sources as follows: Glycerol (~ 20, 40, 50, 60, and 70 g/L) and yeast extract (10 and 20 g/L).

### Preculture preparation

For preculture preparation, 1 mL of stock culture in 50% (v/v) glycerol was aseptically transferred to 90 mL of sterile basal culture medium in a 100 mL rubber-sealed serum bottle. The mixture was incubated without agitation at 30 °C for 7 days. The resulting culture showed OD_620nm_ of 7 (2.56 g_CDW_/L) and was used as inoculum for bioreactor experiments.

### Bioreactor set up

**Fig. 3:**
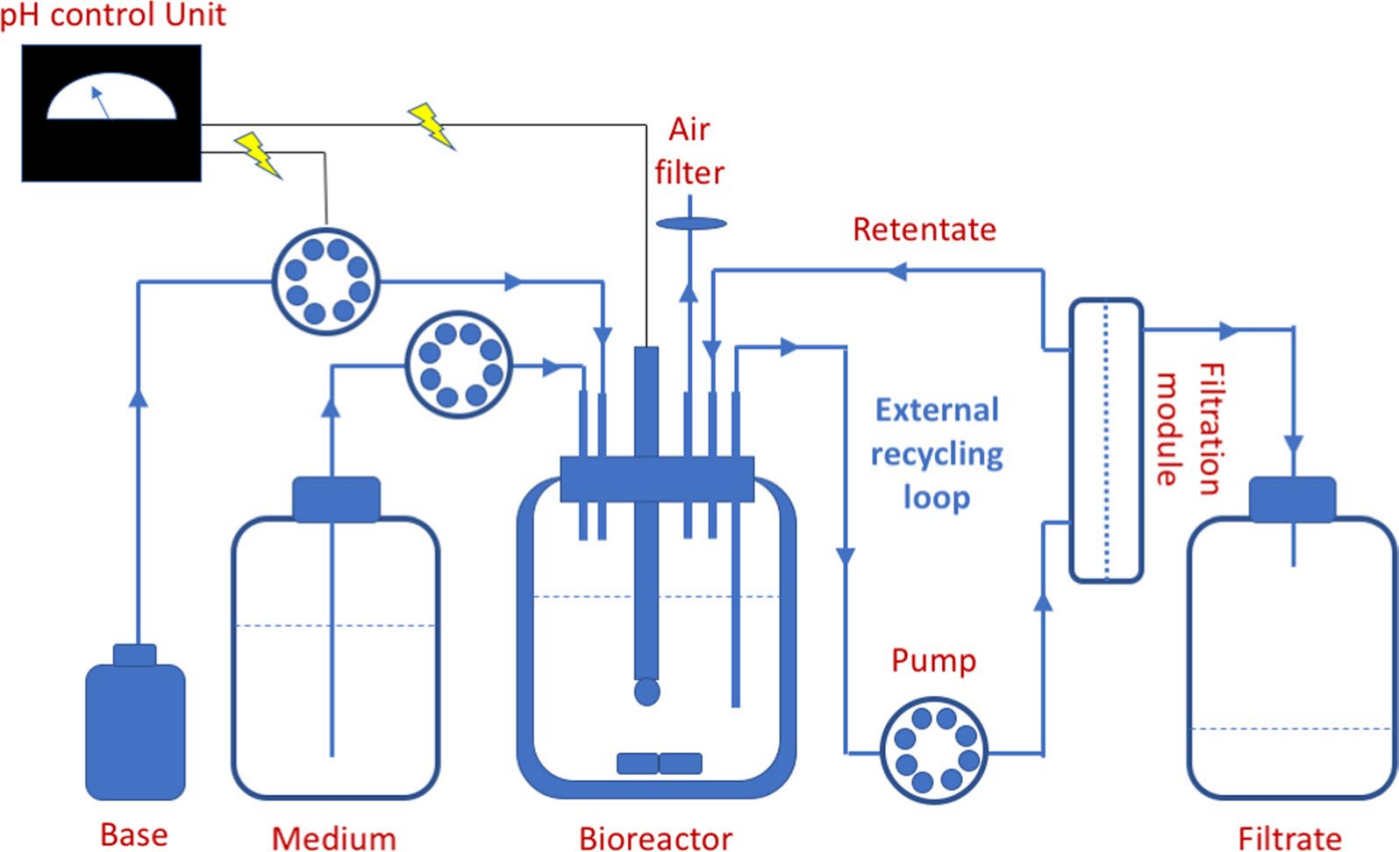
Bioreactor design for continuous propionic acid production with cell recycling. Schematic presentation of the cell recycling fermentation with membrane filter bioreactor set-up used for propionic acid production by *A. acidipropionici*

The bioreactor used for continuous propionic acid production with cell recycling is shown in Fig. 3. It consisted of a 500 mL water-jacketed vessel equipped with a headplate involving ports for pH electrode, base addition, sterile medium addition, sampling, gas release, liquid -outlet, and -inlet for culture recycling to the filtration unit. The vessel was coupled to a tangential flow microfiltration module equipped with a tubular ceramic membrane (0.20 μm pore size, 25 cm length, and 1 cm outer diameter) (Tami Industries, France). The liquid flow in the external recycling loop was controlled by a perstaltic pump (Watson Marlow 604S Ip55, Falmouth, UK), which was manually adjusted to attain a constant volume in the bioreactor (chemostat). During the whole experiment, the temperature was maintained at 30 °C using a circulating water bath, the culture was mixed by stirring at 250 rpm on a magnetic stirrer, and the pH was controlled at 6.5 through the addition of 5 M ammonia via a peristaltic pump controlled by an external pH control unit (Inventron AB, Mölndal, Sweden). Prior to use, the entire reactor set up including 300 mL of basal culture medium was bubbled with oxygen-free nitrogen gas for 15 min and then sterilized by autoclaving at 121 °C for 20 min.

### Continuous production of propionic acid in a bioreactor with cell retention

Thirty milliliters of freshly prepared preculture was aseptically transferred to 300 mL basal culture medium in the bioreactor vessel. The cultivation was started in a batch mode and after glycerol depletion in about 3 days (OD_620nm_ of 13.2), the medium feeding was initiated at a rate of 18 mL/h until the empty spaces in the tubing and external filtration module were filled with the culture and keeping 300 mL inside the bioreactor. Hence, the total working volume reached ≈ 360 mL. At this point, the continuous mode of operation (chemostat) was initiated. With the aim of establishing a steady state with the maximum concentration of glycerol, two dilution rates (D) were evaluated: 0.05 and 0.025 1/h corresponding to a medium feeding rate of 18 and 9 mL/h, respectively. Lowering the dilution rate increases the hydraulic retention time, giving in this way longer time for the cells to consume the nutrients and enhance PA production. Five different glycerol:yeast extract (Gly:YE) mixtures with concentrations of 22.60:10, 43.27:10, 51.40:10, 64.50:10 and 64.50:20 g/L were evaluated at D = 0.05 1/h, while only three mixtures of 62.90:20, 62.90:10 and 70:10 were evaluated at D = 0.025 1/h. Each mixture was operated for at least 4 retention times (≈ 1400 mL). Samples were collected frequently from the bioreactor and the permeate (5 mL each) for measuring the cell density and metabolite concentration, respectively. Bleed out of the culture and increasing the recycling rate had to be applied when required in order to stabilize the system when outflow rate was affected by the cake formation on the membrane surface as a consequence of the increasing biomass.

### Isolation of adapted A. acidipropionici variants

Soon after the continuous fermentation with cell recycling run with Gly:YE concentrations of 70:10 g/L, the bioreactor was run for an additional 3-month period at 30 °C, pH 6.5, and the feed was varied as above between Gly:YE concentrations of 20:10 and 70:10 g/L, and dilution rates of 0.05 and 0.025 1/h. This was primarily done to evaluate the stability of the system and to isolate a potentially adapted mutant. A dense suspension of *A. acidipropionici* cells was withdrawn from the 5-month continuously operated bioreactor, washed twice with sterile saline solution, and then inoculated in serum bottles for isolation of adapted *A. acidipropionici* variants as described ahead. Two sets of media were prepared, the first was designed for isolation of low-pH resistant mutant after three-week cultivation in a 90 mL basal culture medium with the initial pH adjusted to 7.0, 6.0, and 5.0, respectively, and the second set (90 mL basal culture medium supplemented with 10, 20, 30, 40, and 50 g/L propionic acid, respectively, at pH 7.0) for the isolation of PA-resistant variant. Values of OD_620_ > 7 (equivalent to cell dry weight of 2.56 g_CDW_/L), were adopted as a de facto criterion as used earlier in our lab for selecting an adapted strain [29]. After sequential cultivations at low pH and then at high PA concentration, 100 μL of the culture samples were seeded on the surface of a solid medium (with 2% w/v agar adjusted with desired PA concentration and pH), incubated at 30 °C for 7 days to finally isolate adapted bacterial colonies.

### Quantitative analyses

The cell density was followed by measuring the optical density of the culture at 620 nm (OD_620nm_) using UV–Vis spectrophotometer (Ultrospec 1000 (Pharmacia Biotech, Sweden). The cell dry weight (CDW) was determined by centrifugation (Sigma 3-16PK centrifuge) of 5 mL fermentation broth at 4000 g for 20 min in a dried preweighed tube and drying the cell pellet for 12 h at 100 °C in an oven before weighing again and correlating with the volume.

Glycerol, propionic acid, acetic acid, succinic acid, and other minor metabolites were analyzed by HPLC (Jasco) equipped with Aminex HPX-87H organic acid analysis column (Bio-rad, Hercules, California, USA), CTO-6A oven (Shimadzu, Kyoto, Japan), Jasco AS 950–10 intelligent pump, PU 980 automatic intelligent injector (Jasco), and ERC 7515A refractive index detector (ERC, Saitama, Japan). Samples were diluted in MilliQ quality water, acidified with 20% (v/v) H_2_SO_4_ (20 μL per 1 mL of the sample), and then filtered through a 0.45 μm syringe filter prior to analysis. Chromatography was performed using 5 mM H_2_SO_4_ as mobile phase flowing at a rate of 0.6 mL/min, column temperature was maintained at 55 °C, and RI detector temperature at 30 °C.

### Fermentation kinetics

For the batch mode of operation, the fermentation kinetics were calculated as follows:

- *Volumetric production rate: Q*_*P*_* (g*_*P*_*/L.h)* = *[ΔP] / [Δt]*
- *Volumetric biomass productivity: Q*_*X*_* (g*_*X*_*/L.h)* = *[ΔX] / [Δt]*
- *Product yield: Y*_*P/S*_ (g_P_/g_S_) =*| [ΔP] / [ΔS] |*

While, for the continuous mode of operation with cell retention the fermentation kinetics were calculated as follows:

- *Dilution rate: D (1/h)* = *F/V*
- *Retention time: t*_*r*_* (h)* = *1/D*
- *Volumetric production rate: Q*_*P*_* (g*_*P*_*/L.h)* = *D. ΔP*
- *Product yield: Y*_*P/S*_* (g*_*P*_*/g*_*S*_*)* =*|ΔP / ΔS|*

where *P* is the product concentration (g/L), *t* is the time (h), *S* is the substrate concentration (g/L), *X* is the biomass concentration (g_CDW_/L), *F* is the continuous mode flow rate (mL/h), and *V* is the working volume (mL).

## Data Availability

The datasets used and/or analyzed during the current study are available from the corresponding author on reasonable request.
